# Caregiver Burden And Caregiving Benefits in Long-Distance Family Caregivers

**DOI:** 10.1093/geroni/igaf122.3315

**Published:** 2025-12-31

**Authors:** Verena Cimarolli, Francesca Falzarano, Kathrin Boerner

**Affiliations:** LeadingAge, Washington, District of Columbia, United States; University of Southern California, Los Angeles, California, United States; Carl von Ossietzky University of Oldenburg, Oldenburg, Niedersachsen, Germany

## Abstract

A rich empirical knowledge base is available on the impact of caregiving on the well-being of geographically proximate caregivers (CGs) of older adults with chronic illness and on the efficacy of interventions that can support them. These research efforts have largely excluded the about 2 million long-distance family caregivers (LDCs) – CGs living over one hour away from their care recipient (CR). Yet, research comparing LDCs with proximate CGs shows that while LDCs report less physical strain, they experience equal or greater levels of burden and emotional stress. For the development of supportive interventions for LDCs, research identifying factors associated with CG burden and caregiving benefits among this growing group of family CGs is needed. Hence, the purpose of this cross-sectional study (N = 304) was to identify socio-demographic characteristics, primary stressors (e.g., distance from CR, CR cognitive status), and internal resources (i.e., coping, social support, familism) associated with LDCs’ levels of CG burden and positive aspects of caregiving. Two regression analyses examined associations between these factors and burden and benefits. Higher CG burden was significantly associated with being younger, being White, less optimal income adequacy, CR’s worse cognitive status, spending more time helping the CR, and higher actual social support received by the LDC. Higher levels of positive aspects of caregiving were associated with being female, as well as higher levels of problem-focused coping and familism (filial piety). Study findings provide preliminary insights into possible targets of intervention to alleviate caregiving burden and to increase caregiving benefits among LDCs.

